# Feasibility and effectiveness of prone position ventilation technique for postoperative acute lung injury in infants with congenital heart disease: study protocol for a prospective randomized study

**DOI:** 10.1186/s13063-021-05895-1

**Published:** 2021-12-18

**Authors:** Yu-lu Xu, Ya-ping Mi, Meng-xin Zhu, Yue-hong Ren, Wei-juan Gong, Wei-jia Fu, Hui-mei Wang, Lan Ye, Yin Wang, Xiao-yan Zhou, Yan Chen, Yan-yan Chen, Li-qiong Gu, Ying Gu, Bing Jia, Jing Hu, Xiao-jing Hu

**Affiliations:** Children’s Hospital of Fudan University, 399 Wanyuan Road, Minhang District, Shanghai, China

**Keywords:** Congenital heart disease, Prone position ventilation, Pediatric

## Abstract

**Background:**

Prone position ventilation is a widely used lung protection ventilation strategy. The strategy is more convenient to implement in children compared to adults. Due to the precise mechanism of improving oxygenation function, development of pediatric prone ventilation technology has been largely focused on children with acute respiratory distress syndrome. There is a paucity of high-quality studies investigating the effects of prone position ventilation after pediatric cardiac surgery. The purpose of this study is to evaluate the feasibility and effectiveness of prone position ventilation in infants who develop postoperative acute lung injury after surgery for congenital heart disease.

**Methods:**

A single-center, randomized controlled trial of pediatric patients with acute lung injury after surgery for congenital heart disease who will receive prone position ventilation or usual care (control group). A total of 68 children will be enrolled according to the inclusion criteria. The main outcome measures will be lung compliance and oxygenation index. The secondary outcomes will be duration of mechanical ventilation, length of stay in cardiac intensive care unit, reintubation rate, and complication rate.

**Discussion:**

This study will investigate the feasibility and effectiveness of prone position ventilation techniques in children who develop postoperative acute lung injury after surgery for congenital heart disease. The results may help inform strategies to improve airway management after surgery for congenital heart disease.

**Trial registration:**

ClinicalTrials.gov NCT04607993. Initially registered on 29 October 2020.

## Background

Prone position has been used to treat severe hypoxemia in patients with acute respiratory distress syndrome (ARDS) since the 1970s. According to a systematic review, use of the prone position during mechanical ventilation improved survival of patients with ARDS who received protective lung ventilation [1]. Pediatric acute lung injury (PALI) is a common complication of congenital heart disease that presents with refractory hypoxemia. Currently, mechanical ventilation is one of the main methods for the treatment of PALI. Most data regarding management in children are extrapolated from clinical trials conducted in adults or neonates. According to a recent study, only 10% of patients with PARDS receive prone positioning. There are no studies in pediatrics to support regular use of prone positioning in PALI [2]. Prone position ventilation refers to placement of the patient in the prone position during mechanical ventilation to facilitate lung expansion in the atelectatic area and improve the ventilation-perfusion ratio. Critically ill pediatric patients represent a special group, and postural therapy plays an important role in the management of airways of these patients.

Prone position ventilation promotes lung recruitment and improves gas exchange through its effects on pleural pressure and lung compression [2]. Prone position ventilation can reduce the difference between the dorsal and ventral pleural pressure, thereby improving the uniformity of lung ventilation and reducing alveolar hyperinflation and alveolar collapse. This results in improved ventilation and oxygenation, and many patients can maintain this improvement even after switching back to the supine position for ventilation [3, 4]. Studies have demonstrated that prone positioning can significantly improve the oxygenation index of newborns [5], reduce the airway resistance in preterm infants, increase the tidal volume inhaled per unit time, improve the dynamic lung compliance, increase the respiratory efficiency of preterm infants, and reduce the occurrence of apnea [5, 6]. In a study of PARDS, prone position was found to increase the tidal volume and thoracic mobility compared with the supine position [7].

In the supine position, both heart and diaphragm compression may aggravate the collapse of the gravity-dependent area of the lung and worsen hypoxemia and ventilator-related lung injury [8]. During ventilation in the prone position, the heart becomes a gravity-dependent zone, which reduces the pressure on the lung tissue in the middle of the dorsal side. In addition, studies have found no significant effect of prone position on the heart rate, hemodynamics, and cerebral vascular oxygen supply [9].

During mechanical ventilation in the supine position, the effect of posture and sedative muscle relaxants adversely affects the sputum drainage. However, in the prone position, the gravitational force helps improve the sputum drainage [10].

With the advances in cardiac diagnosis and treatment technology, patients undergoing treatment of congenital heart disease tend to be younger and have lower body weight. Maintenance of the respiratory function after cardiac surgery in infants and young children is a key determinant of successful surgical outcomes. Owing to the complexity of cardiac surgery and the prolonged operative time, cardiac surgery is performed under low temperature cardiopulmonary bypass; this increases the risk of hypoxemia after cardiopulmonary bypass. In infants and young children, use of cardiopulmonary bypass increases the pulmonary blood flow and reduces lung tissue compliance. High concentration of oxygen in the medium and the impaired thoracic integrity caused by the surgical incision can easily lead to postoperative hypoxemia [11]. Different degrees of lung injury may occur after cardiac surgery. In our department, the incidence of postoperative pulmonary complications is 11.3%, of which the incidence of atelectasis, VAP, and lung consolidation is 8.2%, 1.4%, and 0.80 %, respectively. Delay in the correction of hypoxemia and lung injury in children may lead to adverse life-threatening outcomes. In such settings, early mechanical ventilation in the prone position can improve the blood oxygen content and alleviate ventilator-related lung injury. At the same time, timely and effective respiratory management and lung physical therapy can be performed to correct hypoxemia. Increase in the partial pressure of blood oxygen can reduce complications and facilitate weaning of the patient from the ventilator. Due to the safety of cardiac surgery, only a few studies [12] have investigated the effect of prone position ventilation for patients with hypoxia after cardiac surgery.

Fineman et al. [13] found no significant differences between the prone position and the supine position in terms of air leakage, enteral nutrition, sedation, or analgesia. Prone positioning caused no hemodynamic instability or arrhythmia; moreover, no serious incidents (such as deintubation) occurred during the process of changing positions. Regarding the start time of prone ventilation after cardiothoracic surgery, Eremenko et al. [14] studied patients who had undergone cardiac surgery with a median chest incision. Prone ventilation was started from 3.6 ± 1.2 days after surgery and lasted for 4–12 days. It was found to significantly improve the pulmonary function and arterial oxygenation within hours. Thus, prone positioning seems to be an effective intervention to improve arterial oxygenation in patients who develop ARDS after cardiovascular surgery.

Studies have shown that prone ventilation is not contraindicated in patients after cardiac surgery [15]. Studies [16] have also shown that prone position ventilation can be used in patients with respiratory failure, ALI, respiratory distress syndrome, and other diseases; moreover, prone position can also be used after apnea, gastroesophageal reflux, and cardiopulmonary bypass surgery. Studies conducted overseas have also demonstrated the feasibility of prone position ventilation in patients after cardiac surgery [17].

Prone position ventilation technology is a widely used lung protection ventilation strategy in clinical settings. Compared with adults, it is more convenient to implement in children. Due to the precise mechanism of improving oxygenation function, development of pediatric prone position ventilation technology is largely focused on pediatric patients with ALI. There is a paucity of high-quality studies investigating the effect of prone position ventilation after pediatric cardiac surgery. Determining the indications for prone position ventilation after surgery and standardization of the process (including the position angle and the prone duration) is a key research imperative. The increasing complexity of surgery for congenital heart disease and the decreasing trend in the age of children undergoing surgery have increased the risk of postoperative pulmonary complications. Therefore, development of prone position ventilation technology for critically-ill children with congenital heart disease is a key imperative in order to reduce postoperative pulmonary complications and shorten the duration of mechanical ventilation.

## Trial objectives

The trial objectives are as follows: to assess the effectiveness and feasibility of prone position ventilation for infants with PALI after surgery for congenital heart disease, to compare the effects of conventional lying position and prone position ventilation on the outcomes of infants with acute lung injury after surgery for congenital heart disease, and to provide a basis for prone position treatment in these children.

## Methods

### Trial design

**Fig. 1:**
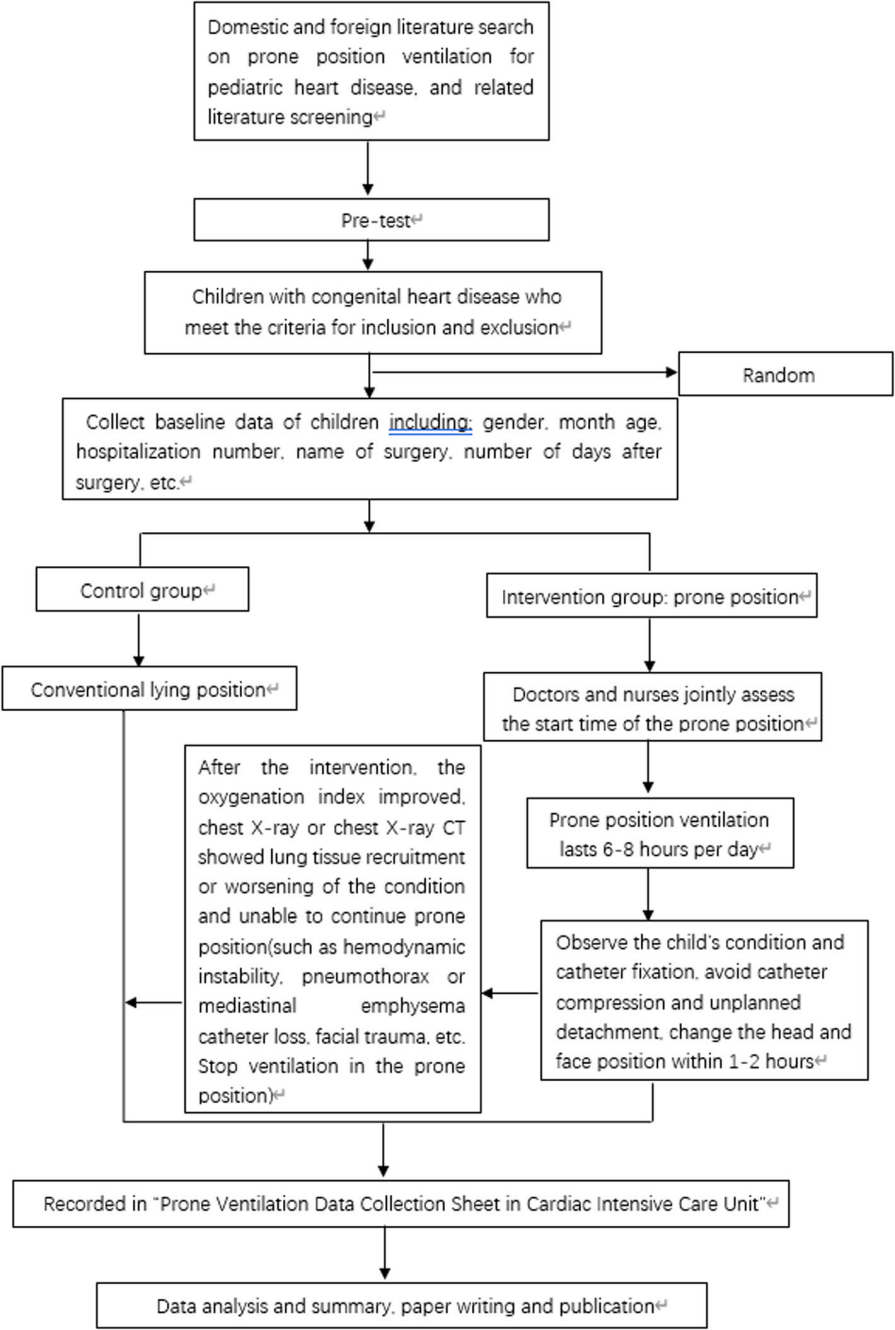
Flow chart providing an overview of important trial steps. First, understand the research situation and methods of this study at home and abroad through the literature, and then conduct a pre-test, correct the research deviation through the pre-test, and randomly group according to the allowance standard, determine the intervention group and the control group, and conduct data collection. Entry and statistical analysis

This study will be a single-center prospective randomized controlled study enrolling infants with postoperative PALI after surgery for congenital heart disease at the Children's Hospital of Fudan University (Fig. 1).

## Participants and recruitment

### Inclusion and exclusion criteria

A total of 68 infants who qualify the following criteria will be included in the study:

1. PALI after surgery for congenital heart disease or patients in whom chest X-ray and CT findings indicate PALI. The diagnostic criteria for PALI: (1) acute onset; (2) hypoxemia, oxygenation index ≥ 4; and (3) chest radiograph showing bilateral lung infiltrates
2. Invasive mechanical ventilation
3. Age range: 0–12 months
4. Patients with stable hemodynamic status, more than 72 h after surgery. Hemodynamic stability defined as Inotropes stable (IS < 10) or based on ECHO (ejection fraction > 50%)
5. Informed consent of family members
6. This study has been approved by the Ethics Committee of the Children’s Hospital of Fudan University, No. (2020) 130

**Table:** 

Inclusion criteria	Exclusion criteria
**- Pediatric patients with lung injury after** surgery for **congenital heart disease or** those in whom **chest X-ray** and **CT** findings indicate the need for augmenting pulmonary drainage due to **pulmonary complications**.	-Hemodynamic instability, severe hypotension, ventricular arrhythmia - Pacemaker dependent - ECMO
**- Invasive mechanical ventilation**	-Intracranial hypertension
**-**Age range: **0–12 months**	-Active acute bleeding
**-Stable hemodynamics, more than 72 h after surgery**	-Spinal injuries and untreated unstable fractures, orthopedic surgery or recent abdominal surgery
**-Informed consent of family members**	-Facial trauma
	Severe pneumothorax
	-Patients with delayed chest closure and wound infection requiring immobilization

### Intervention strategies

1. The family members of the subjects will be informed about the purpose and methods of prone position ventilation, and their consent will be obtained for participation in the study.
2. Careful assessment of the disease status will be performed and adequate fixation of the various cannulas and vascular catheters will be ensured.
3. Enteral nutrition will be suspended 30 min before and the gastrointestinal nutrition tube will be closed before preparing for prone positioning.
4. The timing of start of prone position ventilation will be determined by a team of medical personnel after determining the following: (1) the child has a stable circulation and shows no restlessness, and (2) the routine medical care has been provided.
5. The child will be placed in the prone position with the participation of researchers, doctors, and the nursing staff. The child’s head will be tilted to one side to avoid damage to the eyes and nose due to compression; the arms will be bent upwards and placed on both sides of the head in a W-shaped configuration. The arms can also be placed in another position depending on the specific situation. Both lower limbs will be bent downwards to form an “M” shape; soft pillows will be used below both knee joints to avoid compression.
6. Meticulous care will be exercised to ensure smooth fixation of mechanical ventilation, endotracheal tube, and various catheters.
7. The depth of sedation will be calibrated during the entire prone position ventilation period to achieve good man-machine synchronization.
8. Requirements during prone position ventilation: (1) adequate suction of the sputum and (2) the mechanical ventilation tube is suspended in the air, and the head is in left or right side position for 1–2 h.
9. In a crossover design, the prone position will be maintained for 6–8 h per day until the improvement in oxygenation index (mean airway pressure × FIO_2_ × 100)/PaO_2_). Oxygenation index exceeding the baseline value or chest X-ray or CT findings suggest lung tissue recruitment. Prone position will be discontinued in case of deterioration of the condition (such as development of hemodynamic instability, pneumothorax, mediastinal emphysema, catheter loss, facial trauma, or formation of pressure ulcer).

## Outcomes

### Outcomes (effectiveness)

1. Main outcome indicators: lung compliance, SPO_2_, respiratory rate, peak, positive end-expiratory pressure (PEEP); laboratory indices: oxygenation index (mean airway pressure × FIO_2_ × 100)/PaO_2_), oxygen partial pressure (PaO_2_), carbon dioxide partial pressure (PaCO_2_), pH; chest radiograph.
2. Secondary outcome indicators: (1) duration of mechanical ventilation; (2) length of stay in cardiac intensive care unit (CICU); (3) incidence of reintubation; (4) incidence of complications: pressure skin injury, slippage of the intubation, compression, and displacement of the pipeline; (5) hemodynamic stability: heart rate (HR), invasive arterial blood pressure (ABP), central venous pressure (CVP).

### Sample size/power calculation

According to pre-experimental investigations, the average oxygenation index required by the experimental group of children is 276, with a standard deviation of 68, and the control group has an average oxygenation index of 228, with a standard deviation of 67. Comparing the two independent sample means, *α* = 0.05, *β* = 0.2, the Gpower sample calculation software showed an effect size of 0.71. The ratio of the test group and the control group is 1:1. An attrition rate of 10% has been factored in the sample size calculation. The calculated sample size is 34 patients per group and total sample size is 68 patients.

### Block randomization

1. The block length will be determined along with all possible permutations of the two groups. Setting a block length of 4, all possible arrangements of the intervention group (A) and the control group (B) are AABB, BBAA, ABAB, BABA, BAAB, and ABBA.
2. The sampling numbers will be assigned to each possible permutation block.
3. According to the sample size, the number of blocks will be determined. The sample size of this study is 68, and the number of blocks is 68/4 = 17.
4. The random number function in the computer Excel program will be used to generate 5 integer sequences between 1 and 6, corresponding to the above-mentioned block assignment number; the generated sequence will be numbered and placed in the corresponding numbered sealed opaque envelope.
5. Another researcher (not involved in the implementation of intervention measures) will select the research subjects who qualify the exclusion and inclusion criteria and will open the envelope in the order of the numbers on the envelope.

### Blinding

The research subjects (minors) will have no knowledge of the enrollment status. The research protocol requires informing the children’s family members, so the family members cannot be blinded. The doctor submits the order for prone position ventilation through the doctor’s order system. The doctor’s order system is accessible only to the responsible nurses, so it is not available to the other doctors and nurses. Research nurses who are responsible for data entry and statistics will be blinded to the group identity.

### Statistical analyses

The SPSS 23.0 statistical software will be used for statistical analyses. Quantitative variables conforming to normal distribution will be presented as mean ± standard deviation. Between-group differences will be assessed using the *t* test (paired *t* test before and after prone position, hemodynamic indicators, oxygenation indices, etc., will be assessed using group *t* test). Non-normally distributed quantitative variables will be presented as Chinese numbers (quartiles), and non-parametric tests will be used to assess between-group differences. Categorical variables will be presented as frequency and percentage, and between-group differences will be assessed using chi-squared test or Fisher exact test.

### Data monitoring

The proposed study will be registered with ClinicalTrials.gov. This research project has also been approved by the Fuxing Nursing Research Fund of Fudan University. The organization will monitor and ensure rigor of the entire research process.

### Quality control

The randomization method will be strictly implemented. In the event that the intervention cannot be implemented due to severe intolerance to prone position ventilation or rapid deterioration in a patient’s condition, these patients will be included in the “intention to treat” analysis. All nurses participating in the study intervention will be meticulously trained in order to ensure standardization of the study procedures and data collection. To ensure consistency of data collection, spot checks will be performed to assess the quality of data collected and to take corrective actions to prevent recurrence of errors or omissions.

### Assessment of risks and benefits

All subjects will be closely monitored for adverse events. The incidence of pressure ulcers, wound infection, unplanned rechecks, and catheter slippage will be recorded. These data will be published along with the main results of the study. Personal and telephonic meetings will be regularly conducted to ensure close coordination between the research teams.

### Dissemination

The trial results will be disseminated through research articles published in peer-reviewed journals. We intend to publish 2–3 papers based on our data. Moreover, the findings will be presented in domestic and international conferences and at a seminar conducted at the participating institution.

## Discussion

The purpose of our study was to investigate the feasibility and clinical effectiveness and of prone ventilation in children with ALI after surgery for congenital heart disease in improving oxygenation status. Our findings may help improve the lung protection of critically ill children with congenital heart disease, reduce the duration of mechanical ventilation, and shorten their stay in the CICU. Since this study was conducted for the first time in a single center, there are some deficiencies in the design and content of the study. It is hoped that there will be more in-depth studies on protective lung ventilation in children with congenital heart disease in the future. The results of our study may provide standards for the development of prone position ventilation for children with congenital heart disease. At the same time, we also hope to establish a joint medical-care evaluation mechanism to provide guidance for diagnosis and treatment.

## Trial status

The preliminary experiment is finished; follow-up is ongoing.

## Data Availability

The datasets generated and analyzed during the present study are available from the corresponding author on reasonable request.

## Abbreviations

ARDS: Acute respiratory distress syndrome
PALI: Pediatric acute lung injury
VAP: Ventilator-associated pneumonia
PEEP: Positive end-expiratory pressure
PaO_2_: Oxygen partial pressure
PaCO_2_: Carbon dioxide partial pressure
CICU: Cardiac intensive care unit
HR: Heart rate
ABP: Arterial blood pressure
CVP: Central venous pressure
